# EACVI survey on the evaluation of infective endocarditis

**DOI:** 10.1093/ehjci/jeaa066

**Published:** 2020-05-03

**Authors:** Espen Holte, Marc R Dweck, Nina Ajmone Marsan, Antonello D’Andrea, Robert Manka, Ivan Stankovic, Kristina H Haugaa

**Affiliations:** j1 Clinic of Cardiology, St. Olavs Hospital, PO Box 3250, Torgarden, 7006 Trondheim, Norway; j2 Department of Circulation and Medical Imaging, Norwegian University of Science and Technology NTNU, Trondheim, PO Box 8905, 7491 Trondheim, Norway

**Keywords:** endocarditis, echocardiography, nuclear imaging, CT, survey, EACVI

## Abstract

**Aims:**

To evaluate the diagnosis and imaging of patients with suspected endocarditis and the management in routine clinical practice across Europe, the EACVI Scientific Initiatives Committee performed a survey across European centres. In particular, the routine use of echocardiography, advanced imaging modalities and multidisciplinary team was explored.

**Methods and results:**

A total of 100 European Echocardiography Laboratories from 29 different countries responded to the survey, which consisted of 20 questions. For most of the use of echocardiography and advanced imaging, answers from the centres were relatively homogeneous and demonstrated good adherence to current recommendations. In particular, two-thirds of centres report the use of a specific endocarditis team for decision-making. Echocardiography plays a key role in the diagnosis and management of endocarditis. Nuclear imaging modalities are broadly available among the centres and are mainly used in prosthetic valve endocarditis and cardiac device-related infective endocarditis. Computed tomography (CT) is widely available and used to assess for structural valve abnormalities, neurological complications, and to preoperative assessment of the coronary arteries. Most institutions provide structured patients follow-up following hospital discharge.

**Conclusion:**

In Europe, a relatively homogenous adherence to current recommendation was observed for most diagnostic and management including the follow-up of patients with endocarditis. Decision-making is most commonly performed by a multidisciplinary team. Echocardiography remains the first line and central imaging modality for patient diagnosis and assessment, but 60% of centres also commonly use CT, whilst positron emission tomography imaging is used in patients with prosthetic valve endocarditis or device infection.

## Introduction

Endocarditis is a serious and potentially life-threatening disease, representing a significant burden to the health system. Despite optimal care, mortality approaches 30% at 1 year. An accurate diagnostic workflow is essential to help facilitate early detection and the initiation of appropriate treatment. Both transthoracic (TTE) and transoesophageal echocardiography (TOE) have a well-established role in patients with endocarditis, but recently other imaging modalities including computed tomography (CT) and positron emission tomography (PET) have also demonstrated their utility.

European guidelines for the management of patients with endocarditis have recently been updated^1^: the Task Force for the Management of infective endocarditis of the European Society of Cardiology (ESC), endorsed by European Association for Cardio-Thoracic Surgery (EACTS) and the European Association of Nuclear Medicine (EANM). This document aimed to provide clear and simple recommendations, assisting healthcare providers in their clinical decision-making in patients evaluated for endocarditis. This guideline enhanced the role of PET and CT imaging for the detection of infectious foci when echocardiography remains inconclusive and introduced new diagnostic criteria that included these advanced imaging techniques to improve diagnostic sensitivity. However, there are concerns about the availability of these imaging modalities across different parts of Europe and more generally there remains a lack of randomized controlled trials to guide practice in endocarditis.

The aim of this survey from the European Association of Cardiovascular Imaging (EACVI) Scientific Initiatives Committee was therefore to evaluate the diagnosis and imaging of patients with suspected endocarditis and how they are managed in routine clinical practice across Europe. The EACVI Scientific Initiatives Committee network include imaging centres across Europe and all over the world^2^ and conducts surveys to explore imaging-related management of patients.^2–4^

## Methods

The present survey was conducted by the EACVI Scientific Initiative Committee from 19 December to 28 December 2019 according to the criteria previously described^2–4^ (www.escardio.org/eacvi/surveys). A total of 160 imaging laboratories, mainly based in Europe, were invited to complete an online survey investigating the diagnostic workup and use of imaging in patients with endocarditis and how these patients are managed in routine clinical practice. The survey consisted of 20 questions aimed at understanding the available facilities and workload of each centre, and the preferred imaging strategy including the use of CT and PET imaging.

## Results

In total, 100 (63%) centres from 29 different countries responded to the survey. Responding centres were located in: Austria (1), Belgium (4), Croatia (2), Denmark (6), Ecuador (1), Egypt (1), Finland (3), France (5), Germany (4), Greece (1), Hungary (1), Italy (9), Lebanon (1), Lithuania (2), North Macedonia (1), Malta (2), Netherlands (5), New Zealand (1), Norway (16), Poland (2), Portugal (3), Slovenia (6), Spain (11), Sweden (3), Switzerland (1), Turkey (1), UK (3), and USA (1). Most centres were tertiary centres or University Hospitals, providing a high-volume service (30% of centres performed >300 TTE’s per week whilst only 11% of centres did ≤100 TTE’s per week). Ninety percent of the centres had surgical facilities on site.

### The endocarditis team

**Figure 1:**
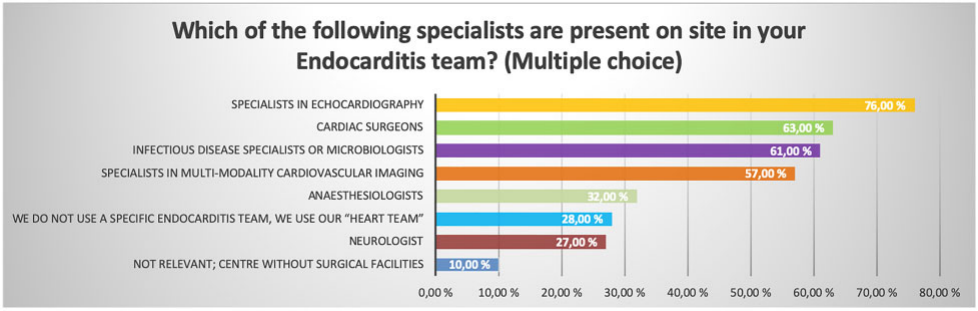
Bar chart showing the specialists present in the endocarditis team among the centres.

Sixty-two percent report the use of a specific endocarditis team for decision-making, while 28% make use of the regular heart team. When present the endocarditis team comprises a specialist in echocardiography (76%), a cardiac surgeon (63%), a specialist in infectious disease or microbiology (61%), and a specialist in multi-modality cardiovascular imaging (57%). Only 27% of centres incorporate a neurologist as part of the endocarditis team (*Figure 1*).

### Echocardiography

**Figure 2:**
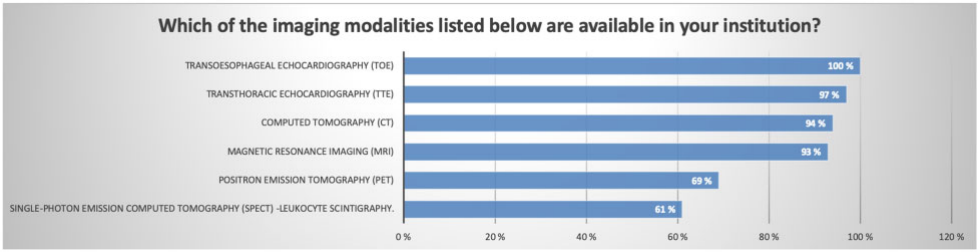
Bar chart showing the available imaging modalities in the different institutions (multiple choice).

TTE and TOE were available at all responding centres, and these modalities remain the first-line imaging assessments for patients with suspected endocarditis. In the specific clinical scenarios proposed in the survey the majority of centres performed both TTE and TOE imaging, although this varied somewhat depending on the specific scenario (*Figure 2*).

In patients with uncomplicated native valve endocarditis confirmed on TTE, only 14% of responders do not perform further imaging assessments. Instead, the large majority (90%) of institutions perform a TOE, and one in 10 centres performed an additional CT (11%) or PET (12%) scan.

In a patient with a persisting high-level of clinical suspicion of native valve endocarditis despite no definitive finding on blood cultures or TTE/TOE, most centres repeated TOE (60%), rather than repeated TTE (16%).

TOE is the preferred modality to evaluate suspected complications such as perforations, abscesses, and fistulae and used in 91% of the centres. Similarly, in patients with a suspected paravalvular leak complicating mitral valve endocarditis on TTE, most centres (88%) performed TOE as the next imaging techniques.

With respect to right-sided endocarditis, most centres (56%) performed repeated imaging with TTE in a patient with *Staphylococcus aureus* endocarditis of their tricuspid valve and moderate regurgitation, although 35% performed follow-up imaging with TOE.

**Figure 3:**
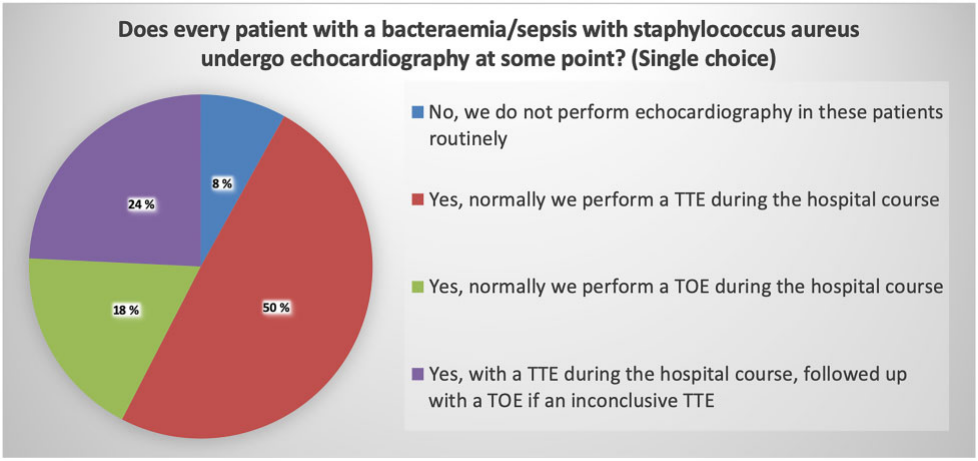
Pie chart showing that most centres perform echocardiographic examinations during a hospital stay in patients with a *Staphylococcus aureus* bacteraemia/sepsis.

Patients with a *S. aureus* bacteraemia but no confirmed diagnosis of endocarditis undergo an echocardiographic examination in most centres (92%) at some point during their hospital stay. Almost half of the centres performed a TTE, while a quarter performed a TTE followed by TOE if required. Of interest, 18% performed TOE as the first diagnostic choice (*Figure 3*).

Finally, with respect to clinical management, a patient with positive blood cultures and a 12-14 mm vegetation on the native mitral valve would undergo surgery within 2–3 days in 16% of centres and during their hospital stay in almost half of the institutions (46%). Forty percent would normally perform no surgery if the patient remains stable without signs of cerebral embolism, heart failure, or uncontrolled sepsis.

### Advanced imaging modalities

The majority of centres have access to advanced nuclear imaging including PET (69%) and single-photon emission computed tomography-leucocyte scintigraphy (61%) (*Figure 2*).

PET is mainly used as a diagnostic tool for prosthesis valve endocarditis (75%) or infection of cardiac implantable electronic devices (71%) when echocardiographic findings are inconclusive. Furthermore, around 20% of centres use PET if echocardiographic techniques are inconclusive in patients with suspected native endocarditis, or blood culture negative endocarditis. Around one-third of centres without PET transfer their patients to a nearby PET-centre for further diagnostic workup. The reason for not transferring patients were mainly high costs, transfer distance, and in some cases, the national unavailability of PET imaging (5%).

Almost all centres have CT (94%) available at their site, with 60% using CT for diagnostic purpose in patients with endocarditis. With respect to valve evaluation, CT is most often used in patients with complex prosthetic valve endocarditis (59%). Moreover, CT is used in a third of centres to assess for perforations, abscesses, and fistulae in native valve endocarditis when TOE is inconclusive (31%).

Patients with confirmed endocarditis and clinical signs suggestive of a neurological complication are evaluated for cerebral consequences with CT as the preferred modality in more than half of centres (57%), whilst magnetic resonance imaging (MRI) is preferred in a third (30%). A fifth of centres routinely investigate for cerebral complications even in absence of neurological symptoms.

When asked how to evaluate the coronary arteries in a 50-year-old patient with no history of ischaemic heart disease and native valve endocarditis requiring urgent surgery, the majority of centres (59%) would use CT coronary angiography, while 30% use invasive angiography. Only a minority of (5%) would proceed to the operating theatre without further examination of the coronaries.

### Discharge and follow-up of patients with endocarditis

Sixty percent of institutions provide written information about antibiotic prophylaxis at discharge, and 30% provide information about thorough oral hygiene in addition to the verbal discussions with the medical team upon discharge. However, 34% of centres have no standardized discharge strategy for patients treated for endocarditis, with the information provided depending on the attending doctor.

The large majority (91%) of centres provide patient follow-up after treatment for endocarditis. Fifty-five percent organize clinical and echocardiographic follow-up within a general cardiology outpatient clinic, while 29% offer follow-up by dedicated personnel: either by the endocarditis team or a heart valve specialist. Only a small minority of centres (9%) do not provide any routine follow-up, but instead, encourage patients to make recontact if symptoms relapse.

## Discussion

The present EACVI survey involved 100 centres from 29 mainly European countries, and reports on the routine clinical practice and diagnostic workup of patients with infective endocarditis. Two-thirds of centres report the use of a specific endocarditis team for decision-making. Echocardiography holds the first line and central position in the diagnosis and management of endocarditis. Nuclear imaging modalities are in fact broadly available among the centres of this survey and are mainly used in prosthetic valve endocarditis and infection of cardiac implantable electronic devices. CT is also widely available and used to assess for structural valve abnormalities, neurological complications, and to assess the coronary arteries prior to surgery. The vast majority of institutions provide structured patients follow-up following hospital discharge.

### The endocarditis team

Endocarditis is often a complex disease process, involving multiple different organ systems. Current guidelines therefore recommend decision-making by an ‘Endocarditis team’ to encourage a multidisciplinary approach to the diagnosis and management of patients with this condition.^1^ Indeed application of an endocarditis team approach has been shown to improve patient outcomes, reducing 1-year mortality in one study.^5^ In this survey, nearly two-thirds of centres reported the use of a specific endocarditis team including a specialist in infectious disease or microbiology. The latter is of increasing importance with growing concerns about antibiotic resistance.

### Echocardiography

Echocardiography was used as the first-line diagnostic approach in all the specific clinical scenarios detailed in this survey. This is consistent with the guidelines.^1^

In patients with uncomplicated native valve endocarditis confirmed with TTE, the large majority (90%) of institutions would still proceed with a TOE for further diagnostic workup. TTE is a highly specific test (specificity 90% similar to TOE), and whilst its sensitivity for the diagnosis of endocarditis has improved (70%), it remains relatively insensitive for associated structural complications. For example, the sensitivity of TTE for abscess formation is 50% compared to 90% for TOE.^6^^,^^7^ The high rate of follow-up TOE imaging following a positive TTE scan for native valve endocarditis is therefore understandable and in harmony with the current guideline recommendations.^1^

Almost all centres would perform repeat imaging in patients with an initially negative examination and a sustained clinical high level of suspicion of an endocarditis. The majority would proceed with echocardiography, 60% and 16% would repeat TOE and TTE, respectively, in accordance with the current guidelines.^1^ In patients with right-sided endocarditis, follow-up imaging is more commonly performed with TTE (56%) although a third would use TOE. TTE usually allows detailed assessment of the tricuspid valve and right ventricle, whilst TOE is more sensitive for pulmonary- and left-sided valve involvement.

Echocardiography is recommended by the current guidelines in patients with *S. aureus* bacteraemia in view of the high frequency of endocarditis in such cases and because of the high morbidity and mortality of *S. aureus* endocarditis.^1^ Consistent with this guideline the majority of centres would perform echocardiography in these patients.

Embolic events have potentially devastating clinical consequences. With rapid diagnosis, initiation of antibiotics and surgery these effects can be minimized. The risk of embolism increases with vegetations >10 mm, although risk stratification remains difficult and surgery is also associated with risk. The exact role of emergency surgery in patients with large vegetations remains controversial. The guidelines provide a Class I level B indication for surgery in patients with a vegetation >10 mm and one or more embolic events.^1^ In our scenario, where the patients had a large vegetation but no clear history of embolism, there was variation in practice: 46% of centres would perform surgery during the hospital stay; one-third within 2–3 days; whereas 40% would not perform surgery in a stable patient.

### The clinical use of advanced imaging techniques

Even though echocardiography remains critical to diagnosis and management of endocarditis the use of other imaging modalities such as CT and PET appears to be increasing. In this survey, almost all centres had access to CT and most centres had access to advanced nuclear techniques.

Consistent with the guidelines, PET imaging is mainly used in the diagnostic work-up of patients with prosthetic endocarditis including graft infections (75%) and infection of cardiac implantable electronic devices (71%) when TTE/TOE findings are negative or doubtful.

CT was also used to assess patients with complex prosthetic valve endocarditis and more generally to detect structural complications of endocarditis (abscesses/pseudoaneurysms, fistulae, etc.) where these are suspected complications and TOE is inconclusive. A trend also commented in the current guidelines.^1^

CT is also used widely to assess for neurological complications and to investigate the coronary arteries in young patients being considered for endocarditis surgery. Fifteen to 30% of patients with endocarditis get symptomatic neurological complications, whereas up to 60% have additional clinically silent cerebral embolisms.^8^^,^^9^ A neurological event may affect the indication of surgery and thereby the management. As expected, most centres evaluate patients with neurological symptoms for cerebral embolism. Of interest, 21% assessed patients for cerebral lesions regardless of neurological symptoms. MRI is also used to assess for neurological complications. It has a higher sensitivity compared to CT but is harder and less feasible to get an urgent MRI scan in clinical practice. This was reflected in the survey.

Invasive angiography, with a potential risk of complications, has traditionally been used to access the coronaries before surgery.^1^ However, CT represents an attractive diagnostic alternative in younger patients, indeed, the majority of centres in this survey would use CT coronary angiography in this situation.

### Follow-up of survivors of endocarditis

Although the risk of recurrence among endocarditis survivors is relatively low (2–6%), patient education and follow-up is important to reduce the risk of relapse and to monitor for potential complications of the index infection. More than half of the centres provided standardized discharge information, with additional written material. This will improve patient education and may increase adherence to advise.

### Limitations

Most respondents were from tertiary centres or university hospitals with a high volume of patients, and the findings of this survey may therefore not be generalizable to other clinical environments.

## Conclusions

In Europe, a relatively homogenous adherence to current recommendation was observed for most diagnostic and management including the follow-up of patients with endocarditis. Decision-making is most commonly performed by a multidisciplinary team including a specialist in infectious diseases or microbiology. Echocardiography (TTE and TOE) remains the first line and central imaging modality for patient diagnosis and assessment, but 60% of centres also commonly use CT, whilst PET imaging is used to investigate patients with prosthetic valve endocarditis or device infection where diagnostic uncertainty persists.
